# PD-1 Antibody Monotherapy for Malignant Melanoma: A Systematic Review and Meta-Analysis

**DOI:** 10.1371/journal.pone.0160485

**Published:** 2016-08-02

**Authors:** Zhijuan Lin, Xing Chen, Zhifeng Li, Yiming Luo, Zhihong Fang, Bing Xu, Mingzhe Han

**Affiliations:** 1 Department of Hematology, the First Affiliated Hospital of Xiamen University, Xiamen, People's Republic of China; 2 Department of Nephrology, the First Affiliated Hospital of Xiamen University, Xiamen, People's Republic of China; 3 State Key Laboratory of Experimental Hematology, Institute of Hematology and Blood Diseases Hospital, Chinese Academy of Medical Sciences and Peking Union Medical College, Tianjin, People's Republic of China; 4 Center for Stem Cell Medicine, Chinese Academy of Medical Sciences, and Department of Stem Cells and Regenerative Medicine, Peking Union Medical College, Tianjin, People's Republic of China; University of Alabama at Birmingham, UNITED STATES

## Abstract

Antibodies targeting programmed death 1 (PD-1) help prevent tumor cells from escaping immune-mediated destruction. We conducted this systematic review and meta-analysis to gain insight into the efficacy of PD-1 antibodies for the treatment of melanoma. Five trials involving 2,828 adult patients were included in this meta-analysis. In patients with previously untreated or refractory melanoma, treatment with PD-1 antibodies significantly improved the six-month progression-free survival (PFS) (HR 0.55, 95% CI 0.50–0.60, P<0.00001) and the overall response rate (OR 3.89, 95% CI 3.12–4.83, P<0.00001). This meta-analysis indicated that anti-PD-1 treatment might provide a significant survival benefit in patients with melanoma. In addition, we found that patients treated with nivolumab reported significantly fewer treatment-related adverse events (OR 0.74, 95% CI 0.57–0.97, P = 0.03) than those treated with other agents, but there was a dose-dependent increase in the frequency of adverse events in patients treated with pembrolizumab.

## Introduction

Malignant melanoma is a type of cancer that develops from pigment-containing cells known as melanocytes. In 2016, an estimated 76,380 new cases will be diagnosed, and 10,130 people will die of melanoma in the United States [1]. A clinical diagnosis of malignant melanoma is confirmed by skin biopsy. Typically, melanoma cells are histologically characterized by the expression of S100, HMB45 and Melan A. The optimal treatment for melanoma remains undetermined, but surgery may be associated with a high cure rate for melanoma in situ. However, patients with high-risk melanoma may require adjuvant treatment, and the prognosis associated with these malignancies is very poor. The estimated five-year disease-free survival rate for advanced melanoma (AM), i.e., stage IIIC and IV disease, is less than 16% [2].

Tumor cells evade immune recognition through multiple mechanisms. One key interaction between cancer cells and the immune system is mediated by programmed death ligand-1 (PD-L1) and programmed death 1 (PD-1) signaling. PD-1 is a member of the CD28 superfamily and is expressed on the surface of activated T-cells and B-cells [3,4]. The human PD-1 gene is located at 2q37.3 and encodes a protein of 288 amino acid residues [5,6]. There are two ligands for the PD-1 receptor, PD-L1 and PD-L2. PD-L1 is mostly present on the surface of hematopoietic and parenchymal cells, whereas PD-L2 is usually present on the surface of macrophages and DCs [7]. PD-1 was first confirmed as a negative regulator of immune responses in a mouse model with a PD-1 null mutation in 1999 [8]. In normal tissue, the combination of PD-1 and PD-L1 protectively inhibits the proliferation of immune cells and induces dysfunction of activated T cells, eventually decreasing autoimmunity and promoting self-tolerance [7]. Upregulation of PD-L1 expression has been reported in many types of tumors, including melanoma, lung cancer, renal carcinoma, and hematological malignancies [9,10]. Binding of PD-L1 to upregulated PD-1 induces apoptosis of tumor-specific cytotoxic T cells and an immunosuppressive effect that promotes tumor cell evasion of immune-mediated destruction [5,6]. PD-1 antibodies inhibit the interaction between PD-1 and its ligands on tumor cells to promote immune-mediated destruction.

PD-1 antibodies have recently emerged as a promising immunotherapeutic approach for the treatment of malignant melanoma, non-small-cell lung cancer, renal cancer cell and hematological malignancies. In a phase 1 study, 296 patients with malignant melanoma, non-small-cell lung cancer, prostate cancer, renal cell cancer or colorectal cancer received nivolumab with different dosages. The rate of PFS at 24 weeks was 30–55% in patients with melanoma and 16–41% in patients with non-small-cell lung cancer [11]. Both nivolumab and pembrolizumab have yielded exciting results for the treatment of different types of malignancies in phase 2 and 3 studies [12–15]. In 2014, pembrolizumab, a humanized IgG4 anti-PD-1 antibody, and nivolumab, a fully human IgG4 anti-PD-1 monoclonal antibody (mAb), were approved in the United States for second- or third-line treatment of patients with AM that was refractory to ipilimumab (BRAF wild-type melanoma) or to ipilimumab and BRAF inhibitors (BRAF V600-mutated melanoma).

To gain further insight into the efficacy and safety of PD-1 antibody treatment, we conducted a systematic review and meta-analysis to compare the efficacy of PD-1 antibody monotherapy with other therapeutic strategies for the treatment of malignant melanoma.

## Methods

This systematic review and meta-analysis was conducted according to the recommendations of the Preferred Reporting Items for Systematic Reviews and Meta-analysis (PRISMA) statement and the Cochrane Handbook (S1 Table).

### Search strategy

We searched the MEDLINE, EMBASE, and Cochrane Library databases without language restrictions. There are three anti-PD-1 agents with the most clinical information: nivolumab, pembrolizumab and pidilizumab. BMS-936558 and MDX-1106 are bynames of nivolumab. MK-3475 is a byname of pembrolizumab, and CT-011 is a byname of pidilizumab. Thus, we used various combinations of the following MeSH terms and keywords to search for studies of interest: anti-PD-1, PD-1 antibody, anti-programmed death 1 antibody, pembrolizumab, MK-3475, nivolumab, BMS-936558, MDX-1106, pidilizumab and CT-011. We also searched for the following medical conditions of interest: melanoma, malignant melanoma and melanomas.

### Inclusion and exclusion criteria

We included all prospective, randomized controlled trials (RCTs) that reported the overall response rate, survival data and treatment-related adverse events associated with anti-PD-1 monotherapy in patients with histologically confirmed melanoma. Melanoma patients who had not previously received systemic treatment for advanced disease and patients with relapsed/refractory disease were included. We excluded all non-comparative, in vitro and animal studies. In addition, we excluded poor quality studies and those with incomplete data or duplicate reports.

### Data extraction and outcomes of interest

The titles and abstracts of all the studies identified in the literature search were screened by two reviewers (Zhijuan Lin and Xing Chen) to verify compliance with the inclusion and exclusion criteria. The same reviewers independently extracted the data and assessed the quality of the publications. Disagreements between the two reviewers were resolved by consensus after a joint second review.

The predefined data extraction form consisted of the publication reference, patient characteristics, the PD-1 antibody regimen [i.e., first-line treatment or relapsed/refractory disease and dosing (mg/m^2^)], the years the trial began and ended, and the chemotherapy regimen used. The following information was also recorded for patients in each treatment arm: number of enrolled patients, median age, female to male ratio, number of events and survival data.

The primary outcome was the six-month progression-free survival (PFS) rate, and the secondary outcomes were the overall response rate and treatment-related adverse events. The overall response rate included complete responses (CRs) and partial responses (PRs). If certain data were not included in the articles, we asked the authors for additional information.

### Quality assessment

The Cochrane Collaboration's tool was used to assess the risks of selection, performance, detection, attrition and reporting biases in the RCTs selected for analysis. Trials with more than two high-risk components were deemed to have a moderate risk of bias, and trials with more than four high-risk components were deemed to have a high risk of bias.

### Data synthesis and analysis

We used Review Manager 5.3 (Cochrane Collaboration) to conduct the meta-analysis. We evaluated the six-month PFS rates using the hazard ratio (HR) and the 95% confidence interval (95% CI). HR values less than 1.0 indicated that anti-PD-1 treatment was associated with a survival benefit and a P-value less than 0.05 was considered statistically significant. We also assessed the overall response rate and treatment-related adverse events using the odds ratio (OR) and the 95% CI. An OR greater than 1.0 indicated a favorable overall response rate in the anti-PD-1 group or a greater incidence of treatment-related adverse events in the anti-PD-1 group; P-values less than 0.05 were considered statistically significant. If significant heterogeneity was observed, the random-effects model was used. In all other cases, the fixed-effects model was used.

## Results

### Description of studies

**Fig 1:**
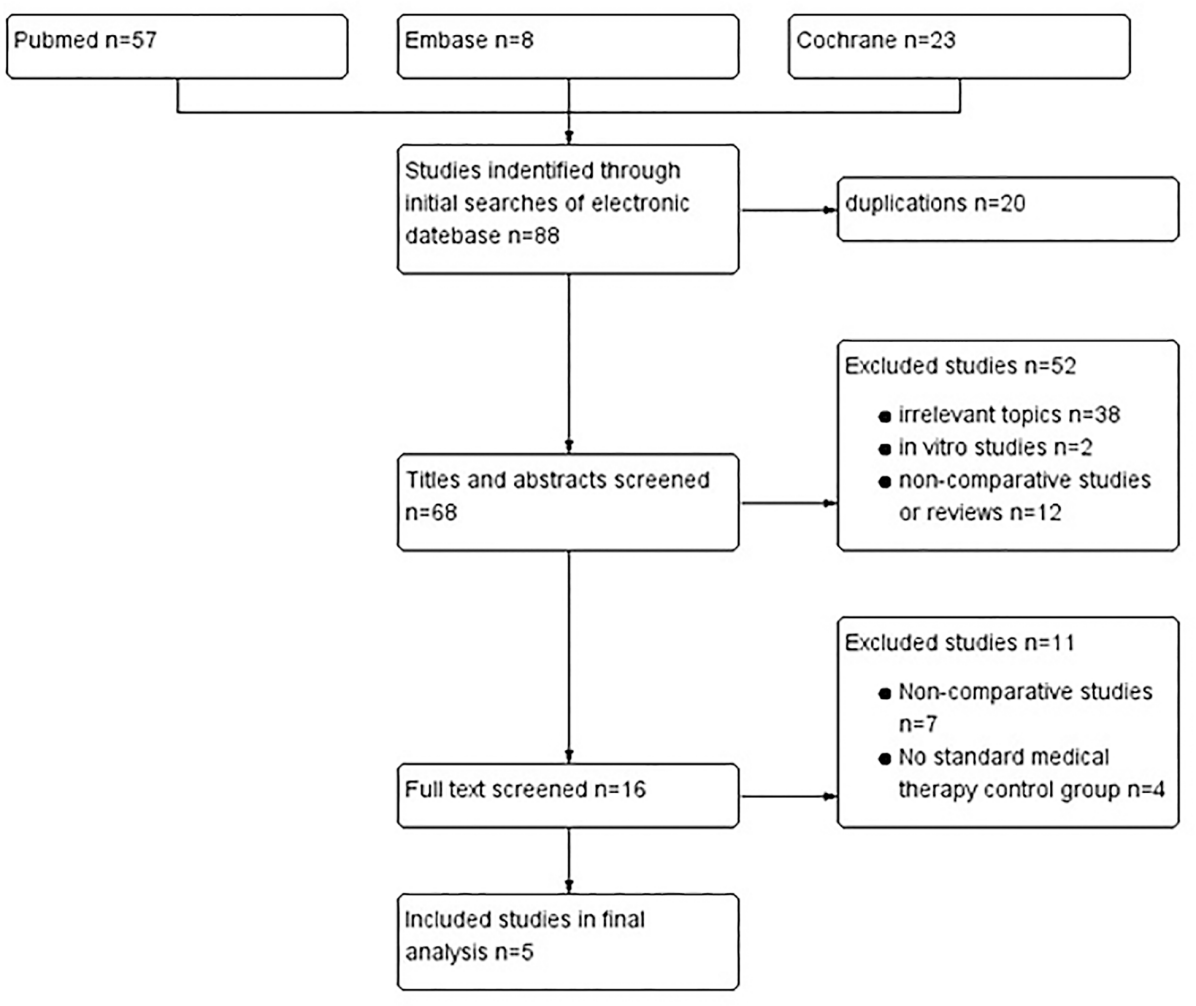
Flow diagram of the literature search.

The literature search identified a total of 88 publications (57 studies from PubMed, 8 from Embase and 23 from Cochrane), and five of the reported trials fulfilled our selection criteria [16–20] (Fig 1). These five trials were multicenter RCTs that included a total of 2,828 patients (1,715 randomized to PD-1 antibody treatment and 1,113 to other treatments). Additional details regarding these five clinical trials are provided in Table 1. In all eligible trials, patients were 18 years of age or older and had histologically confirmed Stage III or IV melanoma. In two trials, patients were previously untreated (n = 2, corresponding to 1049 patients) [16,18]. In the other three trials, patients had progression after anti-CTLA-4 treatment (n = 2, corresponding to 945 patients) [17,20] or had received no more than one previous systemic therapy including chemotherapy, immunotherapy, BRAF or MEK inhibitor (n = 1, corresponding to 834 patients) [19]. Patients in the experimental groups were exposed to nivolumab or pembrolizumab with different dosages, while patients in the control groups received ipilimumab or chemotherapy.

**Table 1:** Descriptive summary of the included patients and randomized trials.

Study	ClinicalTrials.gov number	Recruitment period	Median duration of follow-up (months)	Median age (range)	Patient sex (M/F)	Disease stage	First-line therapy	Intervention		Overall response rate (%)		Median progression-free survival (mo.)	
								PD-1 group	Control group	PD-1 group	Control group	PD-1 group	Control group
Larkin J 2015 [19]	NCT01844505	2013–2014	12.2–12.5	60(18–90)	404/227	stage III or IV	Yes	Nivolumab 3 mg/kg IV every 2 weeks	Ipilimumab 3 mg/kg every 3 weeks; 4 doses	43.7	19.0	6.9	2.9
Ribas A 2015 [17]	KEYNOTE-002	2012–2013	6–14	62(18–89)	327/213	stage III or IV	No (ipilimumab-refractory)	Pembrolizumab 2 mg/kg or 10 mg/kg IV every 3 weeks	Investigator-choice chemotherapy	23.3	4.5	3.7 (2 mg/kg group) 5.4 (10 mg/kg group)	2.6
Robert C 2015 [18]	NCT01721772	2013–2014	5.2–16.7	65(18–87)	246/172	stage III or IV	Yes	Nivolumab 3 mg/kg IV every 2 weeks	Dacarbazine 1000 mg/m^2^ every 3 weeks	40.0	13.9	5.1	2.2
Robert C 2015 [19]	NCT01866319	2013–2014	6.1–11.5	62(18–88)	497/337	stage III or IV	Y/N (received no more than one previous systemic therapy)	Pembrolizumab 10 mg/kg either every 2 or 3 weeks	Ipilimumab 3 mg/kg every 3 weeks; 4 doses	33.2	11.9	5.5 (2-week group) 4.6 (3-week group)	2.8
Weber J 2015 [20]	CheckMate 037	2012–2014	5.6–11.2	59(23–88)	261/144	stage IIIC or IV	No (progression after anti-CTLA4 treatment)	Nivolumab 3 mg/kg IV every 2 weeks	Investigator-choice chemotherapy	31.6	10.6	4.7	4.2

### Risk of bias analysis

**Fig 2:**
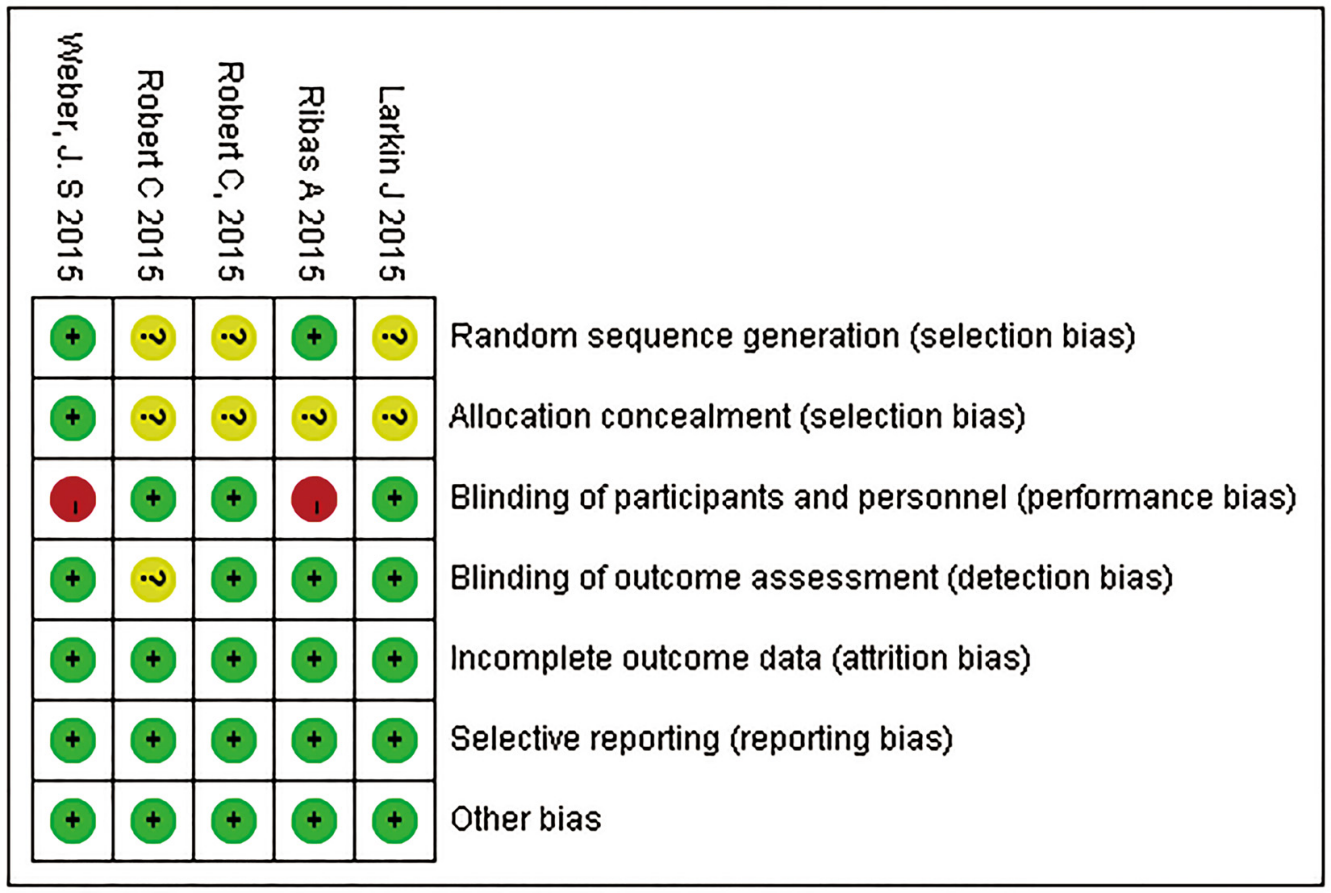
Risk of bias summary. The overall risk of bias was rated as low in all eligible studies.

**Fig 3:**
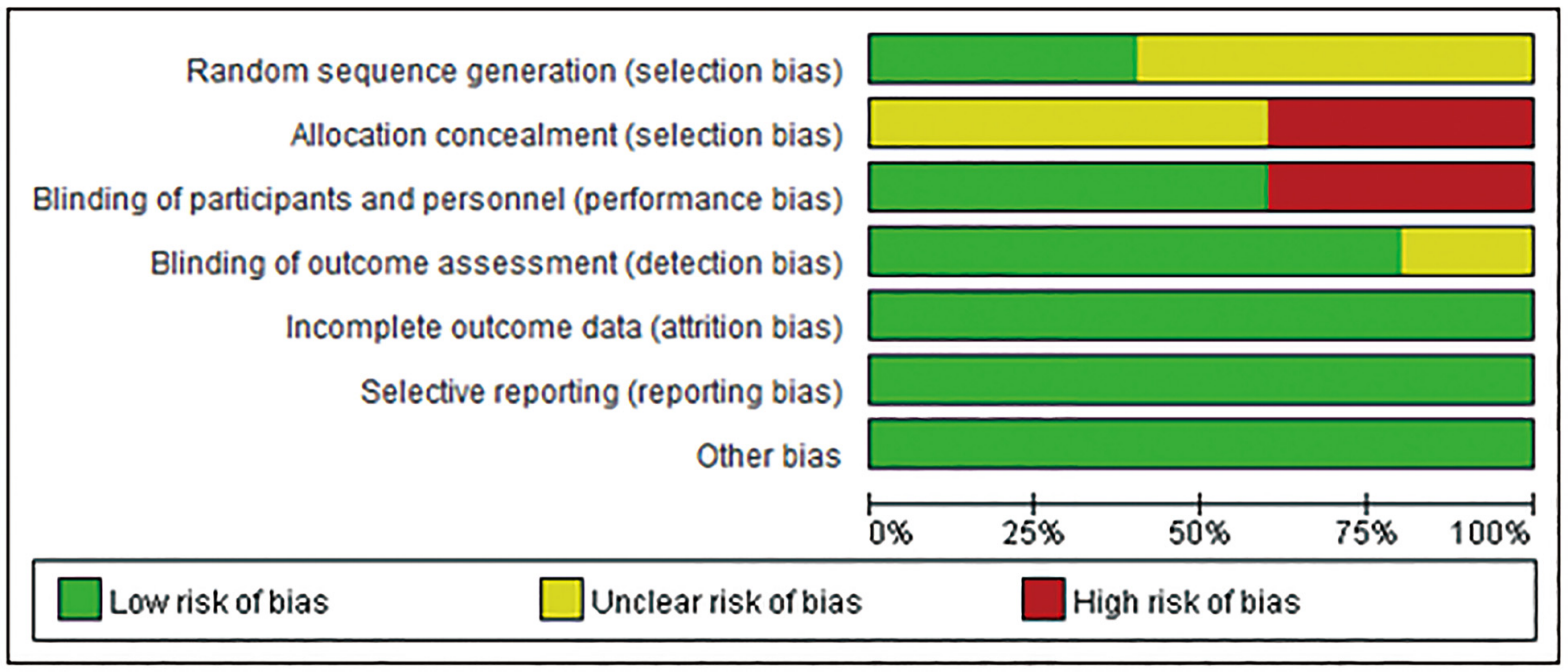
Risk of bias graph. The overall risk of bias was rated as low in all eligible studies.

Based on the Cochrane Collaboration tool, the risk of bias was rated as low in all eligible studies (Figs 2 and 3). Two of the five RCTs were open-label studies. Information regarding random sequence generation and allocation concealment was not provided in three and four RCTs, respectively.

### Efficacy of anti-PD-1 agents

#### Six–month progression-free survival rate

According to Table 1, anti-PD-1 agents prolonged median PFS. The median PFS was more than 4.7 months in the nivolumab group and more than 3.7 months in the pembrolizumab group (Table 1). A high dosage or short intermission of pembrolizumab extended the median progression-free survival.

**Fig 4:**
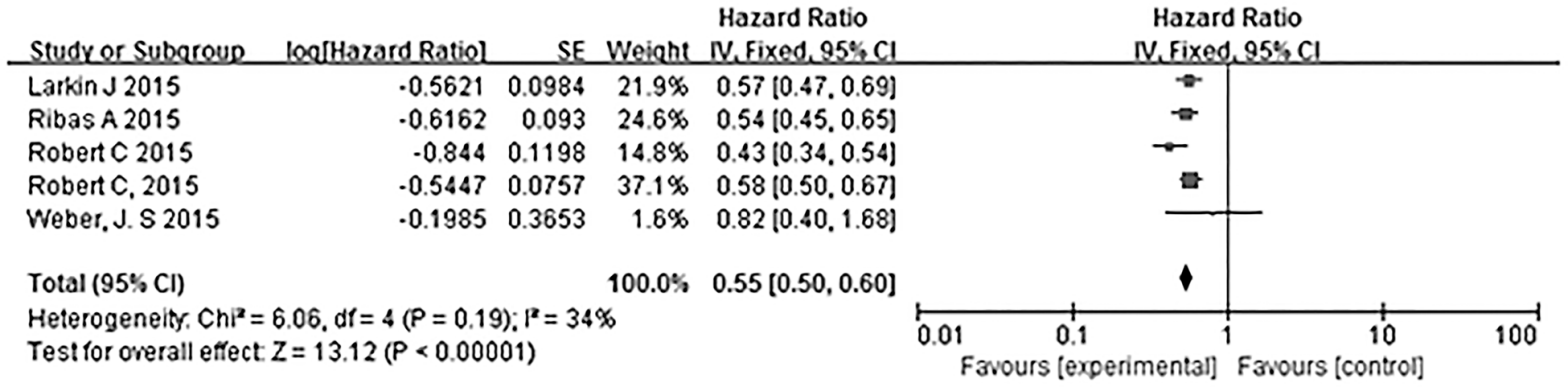
Meta-analysis of the 6-month PFS rates in the PD-1 antibody monotherapy groups and the other groups. The six-month PFS rate was greater among patients who received PD-1 antibodies than among those who received other treatments.

Fig 4 summarizes the comparisons of the six-month PFS rates among the different trials. Overall, patients who received PD-1 antibodies had a significantly greater six-month PFS rate than those who received other treatments, such as chemotherapy and ipilimumab (HR 0.55, 95% CI 0.50–0.60, P<0.00001) (Fig 4). Heterogeneity among the trials was not statistically significant (χ^2^ = 9.06, P = 0.19, I^2^ = 34%). Weber et al. reported differences between the experimental groups and control groups, but the weight of the trial was too low to meet statistical significance (HR 0.82, 95% CI 0.40–1.68) [20]. In the other four trials, there were significant differences between the experimental groups and control groups.

#### Overall response rate

According to Table 1, anti-PD-1 agents were associated with a higher overall response rate than the control groups for both untreated and relapsed/refractory patients. The overall response rate was greater than 40.0% in patients who received nivolumab 3 mg/kg IV every two weeks as front-line therapy and was 31.6% in patients who received nivolumab at the same dosage after progression from anti-CTLA-4 treatment. Different dosages of pembrolizumab also improved the overall response rate in both untreated and relapsed/refractory patients. In two trials, the overall response rate of pembrolizumab was between 23.3% and 33.2% [17,19].

**Fig 5:**
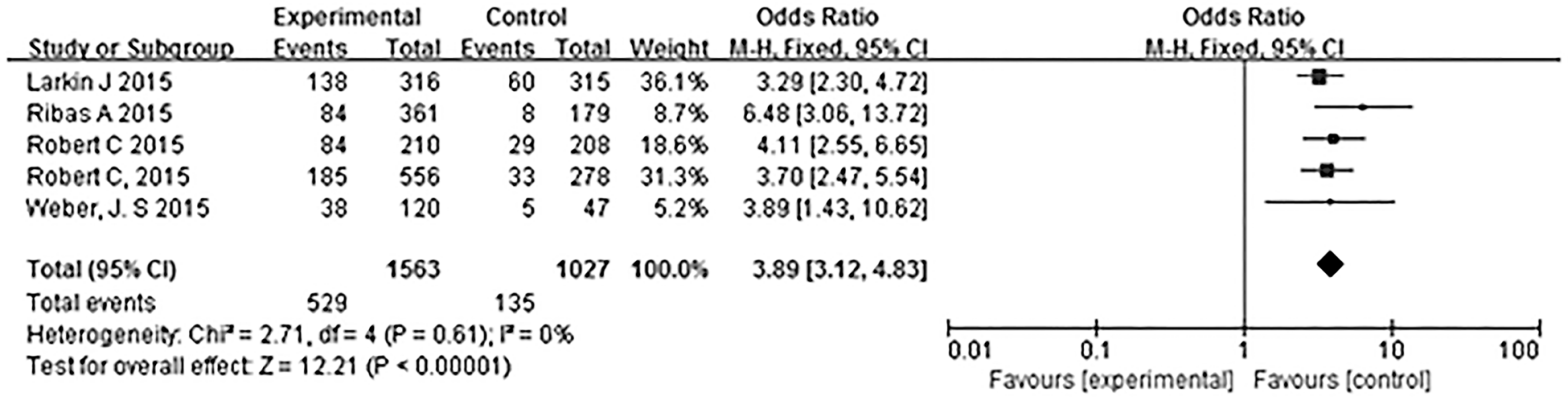
Meta-analysis of the overall response rate in the PD-1 antibody monotherapy groups and the other treatment groups. PD-1 antibody treatment was associated with a higher overall response rate.

Fig 5 summarizes the comparisons of the overall response rates among the different trials. In all five trials, there were significant differences between anti-PD-1 groups and control groups. In the meta-analysis of the five trials, PD-1 antibody treatment was associated with a significantly better overall response rate (OR 3.89, 95% CI 3.12–4.83, P<0.00001) (Fig 5). Heterogeneity among the trials was not statistically significant (χ^2^ = 2.71, P = 0.61, I^2^ = 0%).

### Treatment-related adverse events

**Fig 6:**
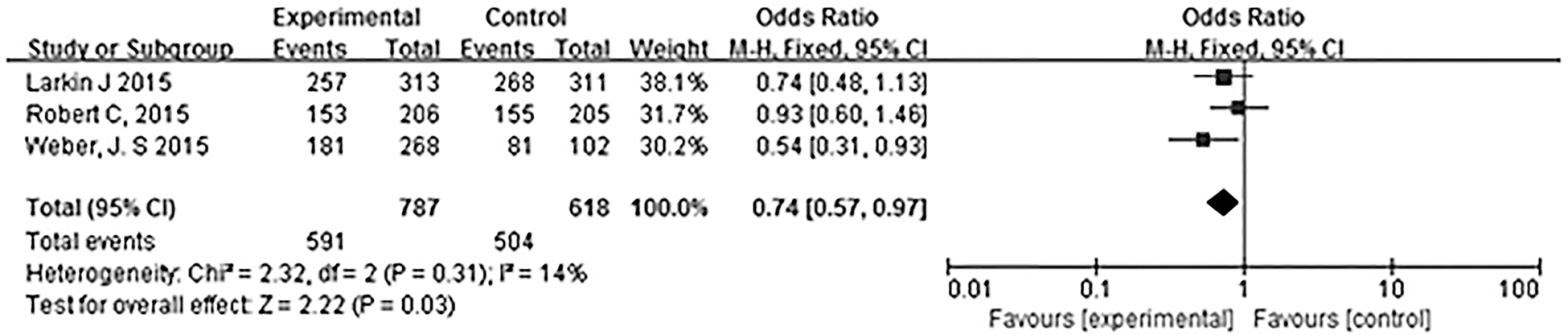
Meta-analysis of the overall treatment-related adverse events associated with nivolumab. Patients treated with nivolumab had a lower incidence of adverse events.

**Fig 7:**
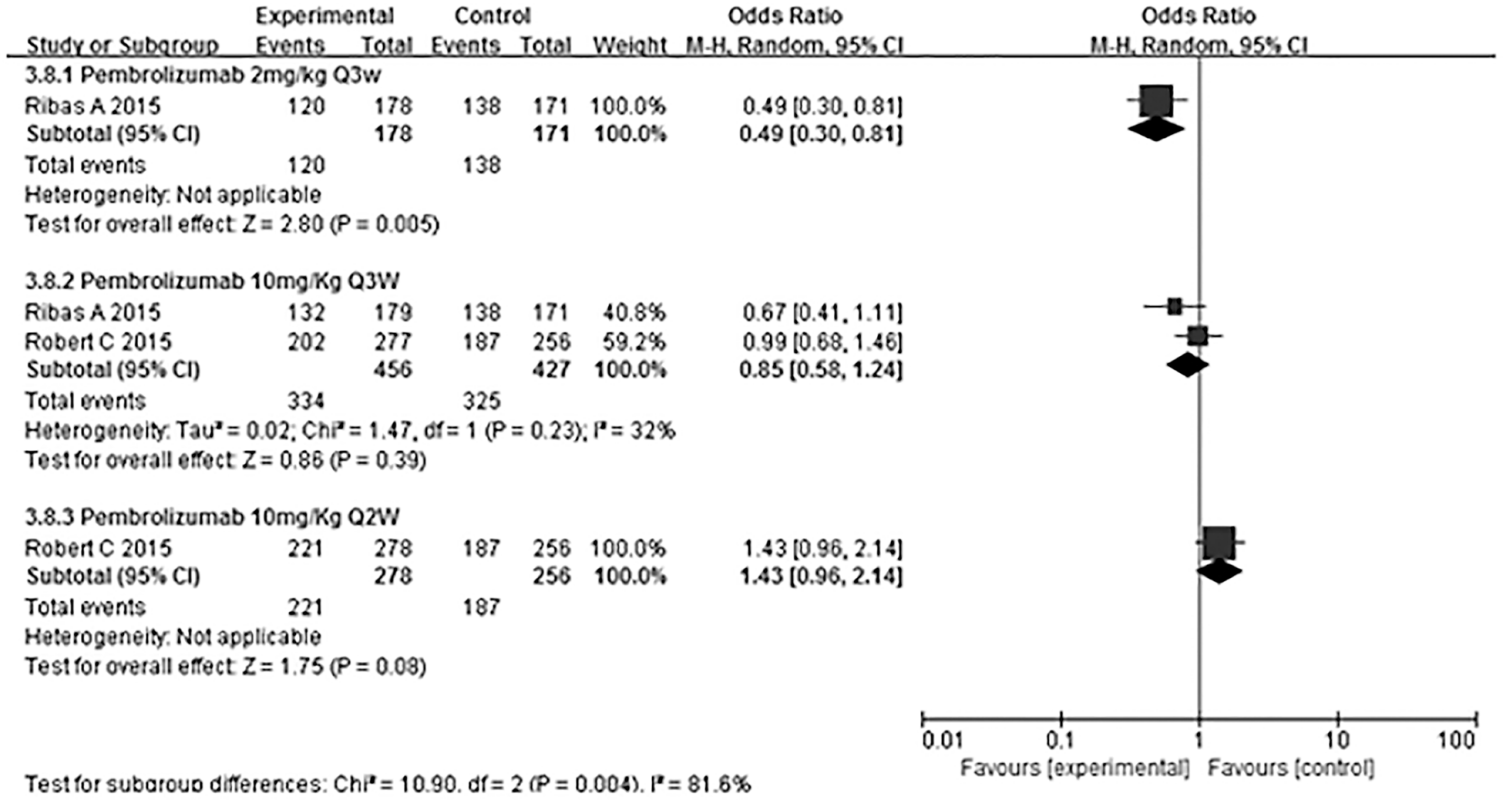
Meta-analysis of the overall treatment-related adverse events associated with pembrolizumab. Subgroup analysis of patients treated with different doses of pembrolizumab revealed a dose-dependent increase in adverse events.

The most commonly reported adverse events associated with PD-1 antibody treatment were fatigue, diarrhea, pruritus, rash and nausea. Table 2 summarizes the treatment-related adverse events in the 5 trials. We also evaluated treatment related-adverse events specific to different PD-1 antibodies and different drug dosages. Larkin et al. [16], Robert et al. [18] and Weber et al. [20] reported treatment-related adverse events associated with nivolumab (Fig 6); specifically, patients treated with this anti-PD-1 antibody reported significantly fewer adverse events (OR 0.74, 95% CI 0.57–0.97, P = 0.03). Heterogeneity among the trials was not statistically significant (χ^2^ = 2.32, P = 0.31, I^2^ = 14%). Ribas et al. [17] and Robert et al. [19] reported treatment-related adverse events associated with pembrolizumab (Fig 7), and a subgroup analysis of different doses revealed a significant dose-dependent increase in adverse events with pembrolizumab (χ^2^ = 10.90, P = 0.004, I^2^ = 81.6%).

**Table 2:** Descriptive summary of treatment-related adverse events.

Study	Intervention in PD-1 group	Adverse events		Intervention in control group	Adverse events	
		Any	Grade 3 or 4		Any	Grade 3 or 4
Larkin J 2015 [16]	Nivolumab 3 mg/kg IV every 2 weeks	257/313	51/313	Ipilimumab 3 mg/kg every 3 weeks; 4 doses	268/311	85/311
Ribas A 2015 [17]	Pembrolizumab 2 mg/kg every 3 weeks	120/178	19/178	Investigator-choice chemotherapy	138/171	45/171
	Pembrolizumab 10 mg/kg every 3 weeks	132/179	25/179	Investigator-choice chemotherapy	138/171	45/171
Robert C 2015 [18]	Nivolumab 3 mg/kg IV every 2 weeks	153/206	24/206	Dacarbazine 1000 mg/m^2^ every 3 weeks	155/205	36/205
Robert C 2015 [19]	Pembrolizumab 10 mg/kg every 2 weeks	221/278	37/278	Ipilimumab 3 mg/kg every 3 weeks; 4 doses	187/256	51/256
	Pembrolizumab 10 mg/kg every 3 weeks	202/279	28/279	Ipilimumab 3 mg/kg every 3 weeks; 4 doses	187/256	51/256
Weber J 2015 [20]	Nivolumab 3 mg/kg IV every 2 weeks	181/268	24/268	Investigator-choice chemotherapy	81/102	32/102

## Discussion

Malignant melanoma develops from pigment-containing cells known as melanocytes. Standard systemic treatment options were previously limited to cytotoxic chemotherapy or interleukin 2 therapy [21]. Dacarbazine, one of the important medications in chemotherapy regimens, is commonly used as monotherapy or in combinations. The median PFS of dacarbazine-treated patients is less than six months, and six-year overall survival is less than 2% [22]. The overall objective response rate of high-dose IL-2 treatment was 16% (95% CI 12%-21%) in a retrospective study of 270 patients [21]. The previous mortality rate for metastatic melanoma was high.

In recent years, the inhibition of melanogenesis has gained wide interest and should be a valid method for anti-melanoma treatment. In normal bodies, melanocytes produce melanin pigments, which can protect the body from solar radiation through their antioxidative and free-radical scavenging actions [23]. Through a series of oxidoreduction reactions, L-tyrosine is transformed into melanin via multiple steps. Tyrosinase is the key enzyme regulating melanin synthesis. The melanocortin/MC1R complex, endothelins, histamine, eicosanoids, sex steroids and vitamin D stimulate melanin synthesis, whereas serotonin, dopamine, acetylcholine and some antagonisms with stimulating agents inhibit melanin synthesis [24]. Nevertheless, in melanoma, the synthesis of melanin could generate an oxidative environment, which would lead to mutations in melanoma, and the capacity of the end-product melanin to scavenge free radicals could generate an hypoxic environment that helps melanoma remain resistant to radio- and chemotherapy [24]. Slominski et al. confirmed that the inhibition of melanogenesis could revert the resistance to chemotherapeutic agents or immunotoxic activity of lymphocytes *in vitro* [25]. Brozyna et al. confirmed that the inhibition of melanin synthesis could increase the sensitivity of melanoma cells to gamma rays *in vitro* [23]. Moreover, melanogenesis could shorten the survival time of patients with metastatic melanoma. Patients with amelanotic metastatic melanomas present a significant longer DFS and OS than patients with melanotic metastatic melanomas [26,27].

Another improvement in melanoma treatment is due to the successful clinical development of therapies targeting the MAPK pathway and immune checkpoint inhibitors that reactivate the anticancer immune response [28]. The three-year overall survival of ipilimumab-treated patients with advanced melanoma is greater than 22% [29].

PD-1 is a cell surface receptor that is expressed on CD4^−^/CD8^−^ thymocytes during thymic development and on activated mature hematopoietic cells, such as T-cells, B-cells, NKT cells and monocytes, after prolonged antigen exposure [30]. The human PD-1 gene is located on chromosome 2q37, and the full-length PD-1 cDNA encodes a 288 amino acid protein [31]. PD-1 binds to its ligands (PD-L1 or PD-L2) on the surface of tumor cells, thus down-regulating T-cell activation and impairing tumor cell recognition. In addition, PD-L1 binding to PD-1 inhibits T-cell proliferation, Bcl-xL (an anti-apoptotic molecule) expression, cytokine production and mTOR pathway activity in immune cells [32,33]. These mechanisms help tumor cells escape immune-mediated destruction. However, anti-PD-1 antibodies inhibit PD-1 binding to PD-L1, thereby promoting T-cell activation and the immune-mediated destruction of tumor cells.

In recent years, PD-1 antibodies have yielded many positive results in tumors, including malignant melanoma, non-small-cell lung cancer, renal cancer cell and hematological malignancies. In an open-label, randomized, phase III study, median overall survival was 9.2 months (95% CI 7.3–13.3) in nivolumab-treated patients with squamous cell non-small-cell lung cancer and 6.0 months (95% CI 5.1 to 7.3) in docetaxel-treated patients. Grade 3 or 4 treatment-related adverse events were only 7% in the nivolumab group versus 55% in the docetaxel group [13]. In non-squamous cell non-small-cell lung cancer, median overall survival was 12.2 months in nivolumab-treated patients and 9.4 months in docetaxel-treated patients. Treatment-related adverse events were also lower in the nivolumab group [12]. In a Phase 1 study of patients with relapsed or refractory Hodgkin's lymphoma treated with nivolumab, the 24-week PFS rate was 86% [15]. In renal cell cancers, breast cancers and ovarian cancers, PD-1 antibodies have also emerged as a promising immunotherapeutic option [14,34,35].

Our meta-analysis evaluated the efficacy and treatment-related adverse events reported in five clinical trials (2,828 cases) that investigated PD-1 antibody treatment in patients with melanoma. Patients who were treated with PD-1 antibodies demonstrated an increased survival rate compared to those who received other treatments, such as chemotherapy and ipilimumab. PD-1 antibody treatment was also associated with an increased overall response rate, regardless of its use as first-line treatment or for refractory/relapsed melanoma. The primary treatment-related adverse events associated with anti-PD-1 antibodies were fatigue, diarrhea, pruritus, rash and nausea. Nivolumab (3 mg/kg IV every two weeks) was associated with significantly fewer adverse events than the other treatment options (OR 0.74, 95% CI 0.57–0.97, P = 0.03). In contrast, pembrolizumab was associated with a dose-dependent increase in adverse events. Overall, in our analysis, anti-PD-1 treatment exhibits efficacy, and the risk of treatment-related adverse events is lower with nivolumab than with the other treatment options.

Another published meta-analysis discusses anti-PD-1 in melanoma. Gandini et al. performed a meta-analysis that evaluated the efficacy of PD-1/PD-L1 antibodies in cancer patients with different PD-L1 expression levels. The analysis of patients with melanoma revealed that PD-L1-positive patients not only present a better objective response rate but also have a lower risk of mortality than PD-L1-negative patients [36]. This meta-analysis reminds us that the PD-L1 status may influence the efficacy of anti-PD-1/PD-L1 therapy, but the analysis did not separately analyze PD-1 and PD-L1 antibodies.

Some limitations of our meta-analysis are worth noting. First, the patients evaluated in this study presented with different stages of disease (untreated or refractory), which may have influenced the efficacy of PD-1 antibody treatment. Second, different chemotherapy regimens were administered to the control groups in different trials; patients in the control groups in two of the five studies were treated with ipilimumab (a CTLA-4 inhibitor), while those in the other studies received chemotherapy. The use of different treatments in the control groups may have affected our assessment of PD-1 efficacy. Third, patients in different studies were treated with different dosages of pembrolizumab; therefore, this analysis cannot provide evidence to support a specific pembrolizumab dosage. Fourth, few trials reported overall survival data, and the follow-up times were not sufficiently long for an analysis of late-stage and fatal complications.

## Conclusion

In summary, our results showed that PD-1 antibody monotherapy significantly improved the survival of melanoma patients with previously untreated or refractory disease. In addition, we found that nivolumab was associated with a lower risk of adverse events, yet there was a trend toward a dose-dependent increase in adverse events with certain PD-1 antibodies. However, the follow-up times of the studies were not sufficiently long to facilitate an analysis of late-stage and fatal complications. We look forward to extending this type of analysis to multicenter, prospective, double-blind and well-designed RCTs to verify the findings of this study and to gain further insight into the efficacy of PD-1 antibody treatment in patients with melanoma.

## Supporting Information

S1 Table PRISMA Checklist. (DOC)
